# Supplementary material for: A feature-guided, focused 3D signal permutation method for subtomogram averaging
Source: J Struct Biol. Author manuscript; Available in PMC 2022 Jun 1. (PMC9149098; doi:10.1016/j.jsb.2022.107851)
Supplement: 1 [file NIHMS1795382-supplement-1.docx]

**A feature-guided, focused 3D signal permutation method for subtomogram averaging**

**John Jacob Peters^†,1,2,3,4,5^, Jeremy Leitz^†,1,2,3,4,5^, Qiang Guo^6,7^, Florian Beck^7^, Wolfgang Baumeister^7^, Axel T. Brunger^1,2,3,4,5*^**

^1^Department of Molecular and Cellular Physiology, Stanford University, Stanford, United States

^2^Department of Neurology and Neurological Sciences, Stanford University, Stanford, United States

^3^Department of Structural Biology, Stanford University, Stanford, United States

^4^Department of Photon Science, Stanford University, Stanford, United States

^5^Howard Hughes Medical Institute, Stanford University, Stanford, United States

^6^State Key Laboratory of Protein and Plant Gene Research, School of Life Sciences and Peking-Tsinghua Center for Life Sciences, Peking University, Beijing 100871, China

^7^Department of Structural Biology, Max Planck Institute of Biochemistry, 82152 Martinsried, Germany

^†^these authors contributed equally

***Correspondence:**

Axel Brunger

[brunger@stanford.edu](mailto:brunger@stanford.edu)

**Keywords:** Cryo-electron tomography, in situ cellular tomography, subtomogram averaging, feature-guided alignment, 3D signal subtraction

**Highlights:**

Feature-guided alignment reveals membrane associated densities

Masked 3D signal permutation enhances subtomogram averages

Masking is important to reveal intermembrane densities

**Abstract**

Advances in electron microscope instrumentation, cryo-electron tomography data collection, and subtomogram averaging have allowed for the *in-situ* visualization of molecules and their complexes in their native environment. Current data processing pipelines commonly extract subtomograms as a cubic subvolume with the key assumption that the selected object of interest is discrete from its surroundings. However, in instances when the object is in its native environment, surrounding densities may negatively affect the subsequent alignment and refinement processes, leading to loss of information due to misalignment. For example, the strong densities from surrounding membranes may dominate the alignment process for membrane proteins. Here, we developed methods for feature-guided subtomogram alignment and 3D signal permutation for subtomogram averaging. Our 3D signal permutation method randomizes and filters voxels outside a mask of any shape and blurs the boundary of the mask that encapsulates the object of interest. The randomization preserves global statistical properties such as mean density and standard deviation of voxel density values, effectively producing a featureless background surrounding the object of interest. This signal permutation process can be repeatedly applied with intervening alignments of the 3D signal-permuted subvolumes, recentering of the mask, and optional adjustments of the shape of the mask. We have implemented these methods in a new processing pipeline which starts from tomograms, contains feature-guided subtomogram extraction and alignment, 3D signal-permutation, and subtomogram visualization tools. As an example, feature-guided alignment and 3D signal permutation leads to improved subtomogram average maps for a dataset of synaptic protein complexes in their native environment.

**Introduction**

Advances in cryo-electron microscopy (cryoEM) instrumentation, data collection, and processing have enabled single particle structure determination at atomic resolution in favorable cases (Nakane et al., 2020). In particular, the development of direct electron detectors has permitted the collection of higher resolution information by improved detective quantum efficiency (DQE), and correcting for electron-beam induced drift has improved resolution (Milazzo et al., 2005; Bammes et al., 2012; McMullan et al., 2014). Single particle cryoEM is mostly performed with purified macromolecules, omitting cellular context, and to study such macromolecules in their native environment, cryo-electron tomography (cryoET), which consists of a collection of tilt series (Eisenstein, 2016; Nogales, 2016), is often used. In principle, the 3D reconstructions obtained from cryoET allow for segmentation and subsequent alignment of characteristic densities such as membranes, filaments, or large macromolecular complexes in their native environment. Subsequently, the resolution of objects of interest can be improved and the effect of the missing wedge alleviated by subtomogram averaging, reaching sub-nanometer resolution in favorable cases (Turoňová et al., 2020; Tegunov et al.; 2021).

Subtomogram averaging generally requires collection of many tomograms. The collection of such large cryoET data sets is often challenging, in part due to the necessary data collection time (up to one hour per tomogram). Improvements in automatic cryoET data collection and beam-image shift have reduced the data collection time (Bouvette et al., 2021), and further advances in instrumentation should ultimately allow continuous cryoET data collection in the future (Chreifi et al., 2019; Eisenstein et al., 2019; Chreifi et al., 2021). Improvements in image processing and subtomogram averaging methods have also resulted in higher quality images of macromolecular complexes in their native environment (Heumann et al., 2011; Galaz-Montoya et al., 2015; Bharat et al., 2015; Bharat and Scheres, 2016; Galaz-Montoya et al., 2016; Turoňová et al., 2017; Tegunov and Cramer, 2019; Wan et al., 2020; Tegunov et al.; 2021). However, even with these advances, subtomogram averaging remains challenging for cases where the object of interest is in a crowded environment. In these cases, many objects may be misaligned by automated procedures leading to lower resolution in best case scenarios, and completely missing or averaging out morphological features in worst case scenarios. Increasing the number of tomograms, and hence object number, is one way to address this issue, but the efficacy of this strategy is fundamentally limited by sample preparation and data collection time. This misalignment issue is particularly vexing for transmembrane proteins in which high-intensity voxels, contributed by the membrane, bias image alignment and classification. Thus, there is a need for development of computational approaches that maximize the desired information about objects of interest that can be obtained by subtomogram averaging from cryoET data sets.

Currently available packages and pipelines for subtomogram averaging (Bharat et al., 2015; Bharat and Scheres, 2016; Galaz-Montoya et al., 2016; Tegunov and Cramer, 2019; Wan et al., 2020; Tegunov et al.; 2021) include CTF determination, CTF correction, and subtomogram extraction, averaging, classification, and 3D refinement. Generally, the classification and 3D refinement functionalities have been adopted from single particle EM approaches where a discrete target object with low-noise surrounding background is assumed. This approach may not be compatible with non-globular continuous objects, such as proteins associated with or embedded in membranes. Simple masking of the 3D class average density maps during refinement only partially addresses the issue of undesirable background information since such masking does not actually remove the voxels from the 3D tomographic reconstructions that are responsible for biasing classification and alignment. Therefore, as refinement progresses, these highly influential background voxels can re-enter the masked region of interest and bias refinement. For single particle cryoEM data sets, signal permutation can be performed by removing the contribution of surrounding densities by partial permutation of such densities in 2D projection images (Morais et al., 2003; Bharat et al., 2015; Nakane et al., 2018). However, it may not be optimal to permute nearby densities in the 2D tilt images of a cryoET data set. Thus, signal permutation in the 3D tomographic reconstruction should be more successful for subtomogram averaging.

Here, we developed a new cryoET/subtomogram averaging pipeline (**Cr**yo**E**T processing for **s**ub**t**omogram **averaging**, CrESTA) that includes feature-guided alignment and a new method for 3D signal permutation for tomographic reconstructions. Our 3D signal permutation method randomizes voxels outside a mask that can be of any shape and that blurs the boundary of the mask. In addition, we present the option to filter the randomized voxels outside the mask to attenuate obvious outliers that may be caused by membrane densities. Our method effectively produces a featureless background surrounding the object of interest in 3D. This signal permutation process can be repeatedly applied with intervening 3D refinements of the 3D signal-permuted subvolumes, recentering of the mask, and optional adjustments of the shape of the mask. CrESTA starts from 3D tomographic reconstructions (including, but not limited to, CTF-corrected tomographic reconstructions using NovaCTF (Turoňová et al., 2017; Wan et al., 2020)) and contains feature-guided subtomogram alignment, extraction, signal permutation, and subtomogram visualization tools. An application to synaptic protein complexes in their native environment illustrates how CrESTA allows for improved processing of subtomogram averaging data.

**Methods**

Here we explored subtomogram alignment strategies and demonstrated the utility of 3D signal permutation. We compared two methods for the initial starting alignment of the subtomograms: free alignment and feature-guided alignment (Figure 1). Moreover, we assessed the effect of a 3D signal permutation method implemented in the NovaCTF/CrESTA/RELION workflow (Figures 2 and 3) on subtomogram averaging.

*Example cryoET data set of synaptic protein complexes*

Synaptic vesicles are neurotransmitter-containing organelles that fuse with the presynaptic plasma membrane of neurons to facilitate chemical neurotransmission. The core of the SNARE protein complex (for Soluble NSF-attachment protein receptor) is composed of syntaxin-1, SNAP-25 (synaptosomal-associated protein 25) which both primarily localize to the plasma membrane, and synaptobrevin/VAMP2 (vesicle-associated membrane protein 2) which primarily resides on the synaptic vesicle. These proteins zipper to generate the energy required to fuse the synaptic vesicle with the neuron plasma membrane. Additional proteins are necessary to ensure fast, precisely Ca^2+^-triggered, fusion in particular: the synaptic vesicle-associated calcium sensor synaptotagmin-1, and soluble accessory proteins Munc-18, Munc-13, complexin, and the AAA+ ATPase NSF and the adapter protein SNAP which act at recycling and priming stages. Elucidating the structure of these proteins in a pre-fusion state is difficult due to their relatively small size (~70 kDa in total for the SNARE complex alone). Previously, crystal structures of a pre-fusion state of the SNARE complex composed of a soluble SNARE mimetic complex that also included a fragment of synaptotagmin-1 and complexin (Zhou et al., 2015, 2017), but these crystal structures lack the full biological context (i.e., full length proteins and membranes). CryoEM/ET provides a means to examine these structures, but current analyses are hindered by the problems discussed above including a crowded molecular environment and non-discrete particles. As an example, we used a cryoET data set of synaptic protein complexes in their native environment between isolated synaptic vesicles (ISVs) and synthetic “SM” acceptor vesicles (simply referred to as “synaptic protein cryoET data set” consisting of 260 tomograms, details in Appendix A). However, our methods are generally applicable to any cryoET data set.

*Tomographic reconstructions*

We tested the two alignment strategies using two methods for tomogram reconstruction and 3D-CTF correction: the previously published RELION (version 3) subtomogram averaging workflow (Bharat et al., 2015; Bharat and Scheres, 2016) and a new workflow that we refer to as NovaCTF/CrESTA/RELION workflow that is similar to STOPGAP (Wan et al., 2020) (Figure 4).

The algorithms for the RELION subtomogram averaging workflow have been previously described (Bharat et al., 2015; Bharat and Scheres, 2016) (Figure 4A). For the synaptic protein cryoET data set we largely used default values for the RELION subtomogram averaging workflow starting with tomographic reconstructions produced by IMOD. Briefly, raw frames were motion corrected using MotionCor2 before being recombined into tilt series using a custom python script (drift_combine.py). Bin 1 and 4 tomographic reconstructions were generated using IMOD. The bin 4 reconstruction was used to manually select object coordinates while objects were extracted by the RELION subtomogram workflow from the bin 1 reconstruction (details in Appendix A).

For 3D signal permutation (described below), a 3D CTF-corrected tomographic reconstruction must be calculated. Moreover, full 3D CTF correction is essential for a tilt series due to the greater than 1 µm defocus difference, or up to 100% error in defocus estimation, in the periphery of the tilt series. We used NovaCTF (Turoňová et al., 2017; Wan et al., 2020) which performs 3D CTF estimation and correction on the entire tomogram. NovaCTF can perform the CTF correction by phase-flipping or by CTF multiplication. For our synaptic protein cryoET data set we used phase-flipping.

We use a parallelized implementation of NovaCTF similar to the STOPGAP implementation of NovaCTF (Figure 4B, details in Appendix B) (Turoňová et al., 2017; Wan et al., 2020). Initial tomograms were reconstructed as above with IMOD, object coordinates selected, and the parameters of the initial tomogram reconstructions were then used to generate a new 3D-CTF corrected reconstruction with NovaCTF. We developed a parallelized bash script (Nova_Updated.sh) that applies *a priori* IMOD alignment transforms and the NovaCTF pipeline to generate CTF-corrected tomographic reconstructions. Nova_Updated.sh first performs defocus estimation using CTFFIND4 with a step size determined by the user, here we used 0.5 µm (Rohou and Grigorieff, 2015) and generates the defocus matrix text file required for NovaCTF defocus algorithm.

The CTF correction implemented in NovaCTF uses the .tlt file generated in the ETOMO GUI of IMOD that contains information about the tilt angles of the images. Here our parallel NovaCTF pipeline then applies the alignments generated in ETOMO using IMOD commands and the fid.xf file that contains the information about the alignment transformations of the images. Next, gold is removed using the erase.fid file, the edges are tapered, if desired, and any views (i.e., specific bad tilt angle images) are removed using either the tilt.com file—which contains an exclusion list of bad images that were excluded in the ETOMO initialization—or a user-generated exclusions.txt file containing a comma-separated list of poor images. Next, the remaining NovaCTF steps are performed: y-z reorientation, filter projections algorithm, and finally the 3dctf algorithm reconstructs the tomogram (Figure 4B) (Turoňová et al., 2017; Wan et al., 2020).

*Extraction of subtomograms*

For our synaptic protein cryoET data set, we performed two separate subtomogram extractions. The first extraction produces synaptic interface subtomograms: that is subtomograms of the entire vesicle-vesicle “synapse”. Synaptic interface subtomograms were extracted using manually selected ISV membrane coordinates, (i.e., the point of the ISV membrane that is closest to the interacting SM vesicle; Figure 1A, asterisk in membrane) for both the RELION subtomogram averaging and NovaCTF/CrESTA/RELION reconstruction workflows. The ISV could be easily identified by the presence of a large protein density belonging to the vesicular ATPase (Figures 3A, 3B). To keep track of and visualize the alignment of the vesicle interfaces, we selected a second point at the center of the ISV (Figure 1A, asterisk in the center of the vesicle).

The second extraction focuses on the individual intermembrane densities (i.e., synaptic protein complex densities) present at the synaptic interface. For this second extraction, coordinates were obtained by visual inspection of Wiener-filtered (Tegunov and Cramer, 2019) synaptic interface subtomograms in Chimera (Figure 3D). The approximate center coordinates of individual intermembrane densities were manually chosen (Figure 3C).

*CTF correction and missing wedge*

The RELION subtomogram averaging workflow includes a 3D CTF correction (Bharat et al., 2015; Bharat and Scheres, 2016) which estimates the defocus using CTFFIND4 (Rohou and Grigorieff, 2015) and corrects the CTF on a per subtomogram basis. 3D CTF corrections are performed for each subtomogram – more precisely, it applies the same CTF correction parameters for all voxels in a particular subtomogram, despite changes in defocus in different portions of the volume (Bharat and Scheres, 2016). The CTF correction is applied in Fourier space, and it also incorporates the effects of the missing wedge and includes a dose-dependent B-factor model (Bharat et al, 2015). For higher tilt images where CTFFIND4 may not produce reliable estimates for some datasets (including our synaptic protein cryoET dataset), the defocus value is set to the average defocus estimate.

For the CTF-corrected tomographic reconstructions generated by NovaCTF, the effect of the missing wedge still needs to be accounted for in subsequent classification and refinement processes with RELION. For our synaptic protein cryoET data set, we generated a synthetic single missing wedge volume for the tilt scheme using *tom_wedge.m* (Nickel et al., 2005) (Figure 3C), and this volume is designated as the “_rlnCtfImage” for each subtomogram in the STAR file inputted into RELION. In principle, this approach can be generalized to also apply dose filtering.

*Initial alignment of subtomograms*

Performing an entirely unbiased, reference-free 3D class averaging with even a modest number of subtomograms is computationally demanding due to the large sample space of possible orientations for each target density. Moreover, such unbiased, reference-free 3D class averaging may not be successful in cases where there is pseudo-symmetry with polarity (see Results). One way to limit this searchable sample space is to first align all subtomograms so that the objects of interest begin with similar orientations. Here we evaluated two methods for this initial alignment: free alignment and feature-guided alignment.

*Free alignment*

We used a two-step approach for initial reference-free alignment of subtomograms. The first step used RELION’s 3D refinement function to align subtomograms with no reference. Subtomograms were aligned and averaged using one class with no reference and loose limitations on translation and no limitations on angular sampling (Figure 1B).

In general, the resulting average 3D map will be in some arbitrary orientation (Figure 1B). To better control subsequent re-alignments (and to compare the results to feature-guided alignment), the initial alignment should be transformed to a standard orientation. For our synaptic protein cryoET data set, the final class average from the initial free alignment was visualized using CrESTA’s “Display mrc in tom_volxyz” tool (Figure 2B). Using this tool, the volume was manually rotated such that the object of interest was arbitrarily aligned to an axis of interest–in the case of an ISV-SM vesicle pair, the axis of closets approach between membranes (i.e., through both vesicles) was oriented to coincide with the z-axis with the ISV membrane on top (Figure 1B dashed line). In the second step, another 3D RELION refinement with one class was performed with this volume as an initial reference. As an alternative, the particles could be directly rotated according to angles determined by the manually rotated object. As a result of this re-alignment step, all subtomograms are oriented such that they can later be rotationally aligned about this axis of interest.

*Feature-guided alignment*

Subtomograms are generally obtained by selecting a single coordinate at the center of the object of interest and then extracting a cubic subvolume around it. This coordinate selection can be done by manual inspection using IMOD (Mastronarde and Held, 2017), other packages such as EMAN2 (Galaz-Montoya et al., 2015; Galaz-Montoya et al., 2016), or obtained by template matching (Hrabe et al., 2012). Naturally, these center coordinates have no orientational information about the object of interest. In cases where additional context about the object’s orientation is available, taking advantage of this information can reduce the degrees of freedom in later refinement steps. Furthermore, in instances of pseudo-symmetry with polarity (e.g., a structure sandwiched between two distinct lipid bilayers), subtomograms may become improperly aligned. To reduce the chances of such misalignment a feature-guided alignment approach may be employed. For example, such an approach has proven useful when examining HIV coat assembly where subtomograms were first aligned based on the geometry of a sphere to generate a starting model (Briggs et al., 2009). We developed a conceptually similar feature-guided alignment approach, where the manual selection of a second coordinate for each object of interest (in addition to the center coordinate), for example a particular membrane that is associated with a membrane protein. Of course, this requires that the identity of the membrane can be unambiguously established by visual inspection. Our approach should be applicable to cellular substructures with contextual information, such as membrane proteins on single membranes or proteins associated with filaments. We then use the vector between the two selected coordinates to align the axis of interest to the arbitrarily selected z-axis. Where *a =* [0 0 1], *v_i_* is the vector between the two manually selected coordinates, *r_i_* is the axis of rotation, and $\theta_{i}$ is angle of rotation for each subtomogram, *i*:

$$r_{i}= \frac{a \times v_{i}}{\left\| a \times v_{i} \right\|}$$

$$\theta_{i}=\cos^{-1}\left( \frac{a \cdot v_{i}}{\left\| a \right\|\cdot\left\| v_{i} \right\|} \right)$$

The rotation matrix R*_i_* is then calculated from the axis of rotation, *r_i_* and the rotation angle, $\theta_{i}$. This rotation matrix is converted to ZYZ intrinsic Euler angles (the RELION convention) using the *scipy.spatial.transform.Rotation.as_euler* package on a subtomogram-by-subtomogram basis. These angles are then set as the “_rlnAngleRot”, “_rlnAngleTilt”, and “_rlnAnglePsi” respectively in the STAR file for each subtomogram. It is important to note that the “_rlnAngleRot” value is randomly assigned at this stage. After feature-guided alignment, we recommend performing 3D classification/restrained refinement, effectively imposing a restrained rotational search around the vector *v*.

For our synaptic protein cryoET data set, this feature-guided alignment produces class average maps that are oriented in the same standard setting as the free alignment method (Figure 1).

*3D signal permutation and filtering of subtomograms*

We developed a 3D signal permutation approach within the NovaCTF/CrESTA/RELION workflow to deemphasize potentially interfering background voxels around an object of interest within a subtomogram (Figure 3). First, a simple geometric binary mask *M* (for example, a sphere or cylinder, depending on the shape of the object of interest) is generated within CrESTA using the “Create mask” module with values set to 1 and 0 inside and outside the mask, respectively. The rotation matrix, *R,* and the translation vector, *T,* from the initial alignment of each subtomogram are inverted and applied to this mask on a per subtomogram basis before the mask, *M*_rot_trans_, is used for 3D signal permutation.

*M*_rot_trans_ = *R^-1^ M - T*

The *cut_part_and_movefunc.m* script then performs the 3D signal permutation on a subtomogram volume, *Vi*, by leaving voxel indices within the *M*_rot_trans_ mask unaffected and randomly permuting the indices of the voxels outside *M*_rot_trans_ mask, resulting in the 3D-signal-permuted volume, *VS_i_* :

*VS_i_* = *V_i_* for voxels *i* inside *M*_rot_trans_

*VS_i_* = *V* _permuted_*_(i)_* for voxels *i* outside *M*_rot_trans_

The permutation of indices of voxels outside the *M*_rot_trans_ mask removes features outside the masked region without changing the global statistics of the voxels of the whole subtomogram (mean and standard deviation). To lessen the impact of the border at the edge of the *M*_rot_trans_ mask, only a randomly selected subset of voxels is permuted outside the mask as a function of distance from the boundary of the mask. The number of randomly permuted voxels is increased as the distance from the boundary of the *M*_rot_trans_ mask increases. Therefore, the information decreases depending on the distance to the boundary of the mask. The problem of calculating a set of voxels which has a defined distance range (pseudo shell) is addressed by “growing” the given mask for a specified amount. The process of growing the mask and voxel permutation is repeated until eventually all voxels outside the grown mask are permuted.

If the background contains voxels that are biasing classification (i.e., extreme values on either end of the voxel intensity distribution), it can be useful to filter these voxels (see Supplementary Figure 1 for example). We included an optional filter that replaces voxels with very large or very small intensities (as identified by user-defined number of standard deviations *w, b* from the mean *m*) with values randomly selected from a normal distribution norm (*m, s*) with a mean, *m,* and standard deviation, *s, (m is usually set to 0 and s set to 1)*.This high-intensity filter maintains the voxel mean and only subtly changes the voxel standard deviation while further deemphasizing contribution from outlier background voxels.

*VS*_filtered_ *_i_* = norm(*m, s*) for voxels with intensity > *w* or < *b* and index *i* outside *M*_rot_trans_

An example of a 3D-signal-permuted subtomogram around an intermembrane density is shown in Figure 3E.

The signal permuted and filtered subtomograms, *VS*_filtered_, can be inputted into RELION or other programs for 3D classification/refinement with the orientation information from initial alignment.

*Repeat 3D signal subextraction*

After convergence of each of the class averages, a new round of 3D signal permutation can be performed. This signal permutation is once again performed on the original subtomograms but uses the alignment information from the previous 3D refinement as opposed to the manually selected coordinates and initial alignments. This step helps correct for inaccuracies in the initial coordinate picking.

Additionally, if the class averages are substantially heterogeneous at this stage, using a unique geometric mask for 3D signal permutation for each class may be beneficial and can be done simultaneously. For example, the class average map obtained from a prior alignment/refinement job could be used to generate a more defined mask. However, it is important to assess potential bias that might be introduced by such masks, for example by cross-validation, e.g., the emergence of new features that are not part of the mask. Subsequent alignment/refinement is performed using these corrected and improved 3D-signal-permuted subtomograms. Depending on the quality of these class averages, this process of subtomogram refinement and unique mask generation followed by 3D signal permutation and 3D refinement can be repeated as many times as desired.

*Pruning classes manually and with cross-correlation and visualization of subtomograms using CrESTA*

For both the RELION subtomogram averaging and the NovaCTF/CrESTA/RELION workflows, throughout the process of alignment, 3D signal permutation, classification/refinement, and all iterations, subtomograms can be visualized using the “Stackbrowser” module in CrESTA. The stackbrowser horizontally tiles individual subtomograms as a series of virtual slices with/without the alignment information applied. The stackbrowser also provides one of two means of pruning subtomograms using CrESTA. Within the stackbrowser, users can deselect poorly aligned/low contrast/misclassified/etc. subtomograms and export selections into a new RELION STAR file. A second pruning approach is within the “Cross-correlation” tab of CrESTA. The “Volume & Star File” module allows users to calculate cross-correlation coefficients between a class average and all the individual subtomograms within a supplied STAR file (with missing wedge information applied in Fourier space) within that class average. Cross-correlation can be focused on a masked region of interest centered around the center of the subtomogram. In our applications we used a cubic mask, but in principle the mask can be of any shape. Additionally, a threshold value can be set to exclude all subtomograms with low cross-correlation coefficients and output a RELION STAR file. Both features offer users greater access to monitoring the progress of refinement and pruning classes.

See Appendix C for an overview of a general implementation of the NovaCTF/CrESTA/RELION pipeline.

**Results**

*Free alignment*

Tomograms of our synaptic complex cryoET data set were reconstructed using a standard reconstruction workflow (Figure 4A). Two coordinates were selected for each ISV-SM interface, the ISV membrane coordinate used for the extraction, and the ISV center coordinate used to assess subsequent alignments (Figure 1A, blue asterisks). More precisely, the ISV membrane coordinate is the position in the ISV membrane that is closest to the SM membrane.

The subtomograms obtained by both the RELION subtomogram averaging and the NovaCTF/CrESTA/RELION workflows (Figure 4) were aligned with no reference (“free alignment”) and no orientational restraints using the 3D refinement function of RELION and were subsequently re-oriented in a standard orientation where the aligned average map consists of a vertically oriented vesicle-vesicle pair along the vector *v* with the ISV density on top (Figure 1B, see also Methods). For better visualization of the alignments, we then superimposed all ISV membrane coordinate at the center of a plane and projected the ISV center points onto this plane for all subtomograms (Figure 5A and F). For the RELION subtomogram averaging workflow, the resulting scatterplot shows ISV centers cluster into two groups—75.6% of center points are in the top cluster and 24.4% are in the bottom cluster—indicating incorrectly flipped configurations of the SM-ISV interface (Figure 5A). In other words, since all ISV center coordinates are expected to belong to the top cluster, the free alignment process yielded a substantial subset of incorrect alignments. In addition to the flipping, there is also some horizontal spread of some ISV centers indicating improper tilting of interfaces.

These initial free alignments were used for 3D classification/*v*-restrained (see Methods) refinement using RELION with five classes (Figure 5C) where the refinement was restrained around the vector *v*. The ISV center orientation was again plotted; it was not improved by 3D classification/*v*-restrained refinement (Figure 5B). For each 3D class average map (Figure 5C), we averaged a 21-pixel thick mid-section slice where the membranes were closest (Figure 5D). We then calculated the average of the center 21 pixels (Figure 5D, demarcated by yellow dashed lines) for each pixel along the vertical axis (Figure 5E). These projections only vaguely suggest the presence of a bilayer as evident by two distinct peaks (red arrows).

Better free alignment was achieved when using the NovaCTF/CrESTA/RELION workflow. There was considerably less flipping after initial alignment compared to the RELION subtomogram averaging workflow (97.9% of center points were in the top cluster and 2.1% in the bottom cluster) (Figure 5F). These alignments were again subjected to 3D classification/*v*-restrained refinement using RELION (Figure 5H) which did not substantially change the spread of ISV center locations (Figure 5G). Projections from class averages yielded stronger evidence for the presence of lipid bilayers, as suggested by double peaks (Figure 5H-J, red arrowheads). Additionally, we measured the angular difference between vectors *v* of the initial alignments from the same sub-volumes after the RELION subtomogram averaging and the NovaCTF/CreSTA/RELION workflows and plotted this difference against the average defocus value determined by CTFFIND4 (Supplementary Figure 3A and B). Nearly all the orthogonal interfaces (i.e., those that have the largest angular differences) in the RELION subtomogram averaging workflow (Figure 5A) arise from tomograms that were taken close to focus. Interfaces that were taken at high defocus were more likely aligned similarly in both workflows. These results suggest that not only is the precision of the CTF estimation an important factor in initial interface alignment, but also important is how the CTF-correction is applied (e.g., phase-flipping and/or Wiener filtering). However, and in-depth analysis of these differences is beyond the scope of what we present here.

*Feature-guided alignment*

We then took the same subtomograms and performed feature-guided alignment using the ISV membrane – ISV center vector *v* (Figure 1C). Here, by design, none of the interfaces are incorrectly flipped or rotated (Figures 5K, P). For both workflows, these rotations were used during subsequent 3D classification/*v*-restrained refinement with RELION (Figures 5L-O and Q-T), yielding five class averages. Only small changes of rotations occurred, and, by design, no flipping, leading to well-aligned ISV centers (Figure 5L and Q). We examined the difference in vector *v* between initial and final alignments and found that after feature-guided alignment, the small rotations observed were not affected by the defocus value (Supplementary Figure 3C and D). However, when comparing the final rotational changes between the RELION subtomogram averaging and NovaCTF/CreSTA/RELION workflows (*i.e.* iteration 10, comparing panels 5L and 5Q), tomograms that were collected closer to focus again produced larger angular changes in the subtomogram alignment (Supplementary Figure 3E and F). Again, this underscores the importance of the precision of the CTF estimation and of the CTF-correction method.

We approximated the membrane bilayer distance as the width at half height of the peaks in the voxel-intensity traces. Additionally, we measured the peak magnitude as the average of the two local maxima within a double peak subtracted by the local minimum within the double peak for each bilayer, where a double peak could be seen (Figure 5E, J, O, and T, red arrowheads). Averages are shown in Figure 5U. Generally, feature-guided alignment with the NovaCTF/CrESTA/RELION workflow led to the sharpest membrane bilayer peaks.

In sum, in all conditions (different workflows and alignments), the ISV vesicle was better resolved than the SM vesicle. This may be related to the larger variation of SM vesicle diameter and curvature than that of ISV vesicles. Yet, none of these alignment procedures yielded class averages with intermembrane densities that could be reflective of synaptic protein complexes. One possible explanation is that intermembrane densities may be too heterogeneously distributed and thus “averaged out” during class averaging when using subtomograms that include a relatively large amount of membrane density. This hypothesis is supported by the fact that individual interfaces in tomographic reconstructions contained clearly visible intermembrane densities (Figure 3).

*3D signal permutation and intensity filtering*

To resolve individual intermembrane densities, we re-extracted subtomograms from NovaCTF tomographic reconstructions around each individual intermembrane density (Figure 3D, right panel). We used the NovaCTF/CrESTA/RELION workflow with feature-guided alignment, followed by 3D classification/*v*-restrained refinement with RELION using a 350 Å diameter spherical mask (Figure 6A). Weak intermembrane density appeared for one class, but this density was small and relatively featureless. Furthermore, this 3D classification/refinement was unstable and collapsed to a single class with no interface density after 60 iterations. We then restricted the RELION mask to an 80-pixel long by 40-pixel diameter cylindrical mask (pixel size = 2.62 Å) and performed 3D classification/*v*-restrained refinement, followed by auto-refinement and post-processing (Figure 6B). This led to the appearance of intermembrane densities for two classes (Class 1 and Class 3). However, bilayers were relatively poorly defined for these two classes. Post-processing and FSC analysis yielded resolutions ranging from 21 to 27 Å (Supplementary Figure 4, black traces).

To better resolve the intermembrane densities (which are presumably synaptic protein complexes), we next performed 3D signal permutation (Figure 3E, Methods) as an intervening step between subtomogram extraction using the same 80-pixel long by 40-pixel diameter cylindrical mask and subsequent 3D classification/*v*-restrained refinement/auto-refinement using a 350 Å diameter spherical mask in RELION. This 3D signal permutation, combined with feature-guided alignment, produced strong densities in all three class average maps at the intermembrane density locations, and each of these intermembrane densities were morphologically distinct from one another (Figure 6C). Class 1 was defined by a ~10 nm long density that is parallel to the lipid bilayers. Class 2 has a simple perpendicular stalk. Class 3 has an ~8 nm perpendicular stalk with a ~10 nm long density that is parallel to the lipid bilayers. Post-processing and FSC analysis resulted in resolutions ranging from 28 to 34 Å (Supplementary Figure 4, magenta traces).

To further improve the quality of our class averages, we next used CrESTA’s cross-correlation coefficient (CCC) tool to remove any subtomograms with CCC values <0. CCC values varied over a wide distribution (Figure 6D). As an example, the class average map from class 3 using subtomograms with CCC values >0 (Figure 6E, left), had sharper bilayer density and more discrete intermembrane densities than subtomograms with CCC values <0 (Figure 6E, right). Negative CCC values might be indicative of high noise or anticorrelations in the particular subtomograms (see Supplementary Figure 2).

CCC filtering was then performed individually for each of the three classes. Additionally, 3D signal permutation was repeated with optimally adjusted cylindrical masks for each class that consider the extent of the interface densities (Figure 7). 3D *v*-restrained refinement/auto-refinement was performed, ultimately yielding the class averages shown in Figure 7A. These refinements stabilized after ~ 200 iterations, and only small changes occurred between 200 and 300 iterations. Post-processing and FSC analysis resulted in estimated resolutions ranging from 24 to 30 Å (Supplementary Figure 4, green traces).

These refined class averages produced interfaces with the greatest morphological detail. In particular, the intermembrane density for classes 1 and 2 showed a more extensive globular and more detailed shape after this procedure. As an example, to validate the class 1 average, we applied the orientations obtained after 300 iterations to subtomograms with CCC values >0 and calculated an average map from the full subtomograms together using these alignments but *without* 3D signal permutation (Figure 7B).

*Discussion and Conclusions*

One of the key limitations to subtomogram averaging can be the low object of interest number, a problem that is compounded by the time-intensive process of collecting tomograms and potential rejection or misalignment of objects. Therefore, optimizing the data processing, subtomogram alignment, and averaging workflow is essential for improving final resolution. Ideally, each object of interest should ultimately be properly aligned and classified to maximize the information content of the resulting class average maps. We developed a new subtomogram averaging workflow (NovaCTF/CrESTA/RELION) that includes 1) feature-guided extraction and alignment of subtomograms around a common axis, 2) 3D signal permutation from subtomograms using geometric and data-inspired masks and the randomization of the surrounding voxel values followed by subtomogram averaging with 3D classification/*v*-restrained refinement, 3) selection of subsets using manual inspection and cross-correlation approaches, and 4) repeat 3D signal permutation and *v*-restrained refinement with realigned/adjusted masks.

Feature-guided alignment of subtomograms from NovaCTF tomographic reconstructions led to reasonably well-defined lipid bilayers in the ISV-SM synthetic fusion system (Figure 5P-T). Lipid bilayer thickness as measure by half-height widths of voxel projections from 3D class averages approached physiological thickness for the NovaCTF/CrESTA/RELION workflow with feature-guided alignment (5.29 ± 0.45 nm average across all bilayers). Furthermore, this approach led to the most distinct ISV bilayer traces having the largest difference of double peak height compared to the local minimum within the double peak. In general, feature-guided alignment was superior to free alignment. Moreover, free alignment performed better on NovaCTF-corrected tomograms versus the RELION subtomogram averaging workflow (97.9% of ISVs were in one cluster versus 75.6%). However, even with the improvements provided by feature-guided alignment, intermembrane densities were only poorly resolved or did not resolve at all.

To resolve such intermembrane densities, we extracted subtomograms around the centers of individual intermembrane densities and performed 3D signal permutation (Figure 3D and E). 3D signal permutation alone led to class averages with better defined lipid bilayers and intermembrane densities after 3D classification/*v*-restrained refinement and auto-refinement (compare Figure 6B and C). Interestingly, even though the class averages produced without 3D signal permutation contained fewer features, resolutions from FSC analysis were higher for these class averages. This is likely because these class averages contained more lipid bilayer which was well aligned and influencing the apparent resolution. Another advantage to 3D signal concerns the missing wedge artifact: voxel randomization outside the mask may reduce the artificial misalignment of subtomograms based on the missing wedge. Moreover, repeat 3D signal permutation with realigned/adjusted masks and added cross-correlation pruning ultimately produced the highest quality 3D maps (Figure 7A), resulting in more extensive and detailed intermembrane densities. In particular, a more pronounced intermembrane density emerged for class 2, whereas for 3D classification/*v*-refinement without 3D signal permutation, there is no observable intermembrane density for class 2 (Figure 6B), and even for 3D refinement with 3D signal permutation prior to realignment and adjustment of the masks, there was little intermembrane density (compare Figures 6C and 7A).

Class 1 might appear as though it could be due to average of intermembrane densities that contain tilting of the synaptic vesicle (top) membrane. However, application of the alignments to calculate an expanded average map without 3D signal permutation reveal that this intermembrane density is distinct from the lipid bilayer. This expanded average map is wholly unobtainable from standard averaging (compare Figure 7B with Figure 6A). The expanded map reveals an elongated intermembrane density (length ~ 100 Å) that is sandwiched between lipid bilayers. We note that the length of the postfusion (*cis*) SNARE complex is of comparable length (Sutton et al., 1998). However, since the SNAREs is expected to be in a *trans* conformation that is presumably shorter, the intermembrane density in Figure 7B likely represents a complex of SNAREs and other factors, including synaptotagmins. Additional tomograms are needed to resolve individual molecules in this density map.

Our NovaCTF/CrESTA/RELION workflow marks a substantial increase in user interactions with subtomogram averaging data processing. CrESTA allows users to filter subtomograms as well as rotate subtomograms based on results from RELION refinement. Beyond this, CrESTA also marks the first time 3D signal permutation from subtomograms can be performed in a manner that de-emphasizes background voxels and maintains an image mean of 0 and standard deviation near 1. Existing image alignment and classification algorithms are designed for globular objects of interest, such as single proteins or protein complexes. Continuous membranes, membrane-membrane interfaces, and densely packed targets are not perfectly compatible with the current algorithms used in single particle analysis, in large part due to assumptions made about the region of interest versus background during solvent noise calculation. CrESTA addresses this problem by altering subtomograms directly to de-emphasize background voxels. Combined with manual inspection, these approaches offer the user greater oversight and control of the refinement process in 3D refinement software packages.

*Broad uses of feature-guided alignment and 3D signal permutation*

One of the major hurdles to resolving a protein complex sandwiched between continuous membranes, as shown here, is the substantial signal produced by the membranes effectively produces a pseudo-symmetric system with polarity that is prone to misalignment. CrESTA allows users to define and apply a known vector to the starting subtomograms, and thus directly addresses this issue. Therefore, in addition to cell-free systems like the synaptic protein complex example presented here, CrESTA and the NovaCTF/CrESTA/RELION workflow should prove useful in other biological systems. For instance, CrESTA would likely be useful in cellular tomography, where objects of interest are more likely to be in feature-rich regions. Additionally, cellular context provides orientation information for objects of interest, and feature-guided alignment will take advantage of this information. For example, our approaches should be generally applicable to all membrane-membrane or cell-cell junctions.

**Funding**

We thank the National Institutes of Health (grant R37MH063105 to ATB) and the National Science Foundation Graduate Research Fellowship (grant 2016205587 to JJP) for support.

**Acknowledgements**

We thank Radostin Danev, Yoshiyuki Fukuda, William Wan, and Jürgen Plitzko for stimulating discussions and help with data collection at the early stage of this project.

*Figure 1. Subtomogram extraction and alignment strategies.*

**(A)** Object coordinates were used to extract subtomograms from tomographic reconstructions. Following extraction, subtomograms were aligned in one of two workflows: **(B)** free alignment or **(C)** feature-guided alignment. **(B)** In free alignment, all subtomograms are first aligned to generate a class average model. The model is then manually rotated to an axis of interest (dashed line). Subtomograms are then aligned to this manually rotated class average model to produce initially aligned particles. **(C)** The Feature-guided alignment makes use of the selection of a second object coordinate, here the ISV center coordinates (blue asterisks in the center of the vesicle). A vector, *v*, between the ISV membrane and center coordinates is then used to align all subtomograms (red arrow, here aligning all vesicle-vesicle pairs with the ISV vesicle on top).

*Figure 2. CrESTA GUI for 3D signal permutation, visualization, subset selection by cross-correlation.*

**(A)** Subtomogram visualization module. **(B)** Mask creation and 3D signal permutation module. **(C)** Cross-correlation module.

*Figure 3. 3D signal permutation of intermembrane densities.*

**(A)** A 2D xy slice through a representative tomographic reconstruction (white arrow heads indicate putative ISV-SM vesicle pairs). **(B)** Segmentation of subtomogram of a representative “synaptic” interface (ISV = isolated synaptic vesicle, SM = synthetic plasma membrane vesicle) observed in a tomographic reconstruction from our synaptic protein cryoET data set. Segmentation was manually performed using Amira (Stalling et al., 2005). The blue asterisk marks a V-ATPase, a useful landmark for feature-directed alignment. **(C)** Missing wedge volume for RELION 3D classification and refinement (also used for cross-correlation calculations). **(D)** Raw original subtomogram (left) of a synaptic interface and the corresponding Wiener-filtered volume (Tegunov and Cramer, 2019) (defocus value of -3.0 and a signal-to-noise ratio falloff of 1.2) (right). Red arrow identifies target protein density between membranes. The subtomogram was extracted with RELION with inverted contrast, a 256-pixel box size (pixel size 2.62 Å), and was normalized. **(E)** 3D signal permutation around an intermembrane density by randomization and intensity filtering (see Methods) of the voxels outside a cylinder mask at the center of the intermembrane density in panel D. The raw (left) and Wiener-filtered (defocus value of -3.0 and a signal-to-noise ratio falloff of 1.2) (right) 3D volumes are shown.

*Figure 4. NovaCTF/CrESTA/RELION data workflow summary.*

General overview of the tomographic reconstruction pipelines using either the (A) RELION subtomogram averaging workflow or the (B) NovaCTF/CrESTA/RELION workflow. Programs and/or the names of specific shell scripts are bracketed. Blue boxes represent custom scripts, red boxes represent scripts specific to the NovaCTF workflow (Turoňová, et al., 2017). In both workflows, after tomogram reconstruction, two methods of initial alignment were used followed by 3D classification.

*Figure 5.* *Feature-guided alignment in NovaCTF tomographic reconstructions results in well-defined ISV membranes.*

**(A-J)** Free alignment of 1473 subtomograms using either RELION or NovaCTF/CrESTA/RELION workflows (see Methods), **(A)** ISV center coordinates were projected onto a plane after rotations obtained by free alignment (blue dots, top scatterplot) cluster into two groups with respect to the ISV membrane coordinate (gray dot), indicating flipped orientations. **(B)** After 3D classification/*v*-restrained refinement with RELION, ISV centers remain clustered into two groups. **(C)** Class average maps resulting from 3D classification/ *v*-restrained refinement. In all cases the ISV vesicle is on top. **(D)** A 21-pixel midsection-slice for each class average from **(C)** normalized to a mean of 0 and standard deviation (S.D.) of 1**.** **(E)** The pixel intensity profile of the center 21-pixels of a midsection-slice (regions marked by the yellow dashed lines in **(D)**) show two distinct peaks indicating a clear bilayer (red arrow heads). Pixel intensity values are standard deviations from the mean. **(F-J)** Panels are the same as **(A-E)** but using the NovaCTF/CrESTA/RELION subtomogram averaging workflow. **(F)** Scatterplot of ISV center coordinates indicates some modest clustering indicating interface flipping, but lateral dispersion, indicating tilting, is limited even after classification **(G)**. **(H-J)** As in **(C-E)** but using subtomograms reconstructed using the NovaCTF/CrESTA/RELION workflow**.** Membrane bilayers, especially ISV membranes, become more prominent. **(K-T)** The same panels as shown above but now using feature-guided alignment. **(K-O)** Subtomograms reconstructed using the RELION workflow. **(K)** Feature-guided alignment, by definition, yields perfectly aligned interfaces as a starting point. **(L)** After 3D classification/*v*-restrained refinement with RELION there is some minimal lateral dispersion, indicating tilting but largely interfaces remain correctly oriented. **(M-O)** as in **(C-E)**. **(P-T)** Subtomograms generated using the NovaCTF/CrESTA/RELION workflow and feature-guided alignment strategy. **(P and Q)** With feature-guided alignment, there is no interface flipping and tilting is largely reduced. **(S and T)** The lipid bilayer of the ISV is well-defined. **(U)** Average peak half-heights and amplitudes for traces in **E**, **J**, **O**, and **T.** For all cases, we performed 3D RELION classification/*v*-restrained refinement with five classes for 10 iterations with a 3.7° initial angular sampling rate, a 5-pixel offset range and 1-pixel offset step, a regularization parameter of 2, and C2 symmetry (around the axis defined by the tilt and psi angles) was imposed. Prior angles were provided for the “tilt” and “psi” angles, and these angles were limited to a 5° local search range, effectively imposing a restrained rotational search around the vector *v*. To restrict these angles, “_rlnAngleTilt” and “_rlnAnglePsi” values were added to the RELION STAR file as “_rlnAngleTiltPrior” and “_rlnAnglePsiPrior” respectively. Continuing iterations led noisier class averages.

*Figure 6. Intermembrane densities at synaptic vesicle interfaces.*

**(A)** 3D classification/*v*-restrained refinement with RELION of 1473 subtomograms extracted from NovaCTF tomographic reconstructions around the centers of individual synaptic protein complexes and feature-guided alignment using a 350 Å diameter spherical mask for RELION refinement. Classes 1, 2, and 3 contain 216, 1035, and 222 intermembrane complexes, respectively. Contour levels of normalized volumes are 3.09, 2.26, and 2.47 σ, respectively. **(B)** Auto-refined and post-processed class averages after 3D classification/*v*-restrained refinement of subtomograms extracted from NovaCTF tomographic reconstructions around the centers of synaptic protein complexes using feature-guided alignment and an 80-pixel long by 40-pixel diameter cylindrical mask for RELION refinement. Classes 1, 2, 3 contain 492, 452, and 529 intermembrane complexes, respectively. Class averages were filtered to the respective resolution calculated by post-processing—26.8 Å, 21.0 Å, and 24.0 Å for class 1, 2, and 3 respectively. Class averages have been cropped using a the 80x40 pixel cylindrical mask. Contour levels of normalized volumes are 6.51, 8.13, and 9.84 σ, respectively. **(C)** Auto-refined and post-processed class averages after 3D classification/*v*-restrained refinement of subtomograms extracted from NovaCTF tomographic reconstructions around the centers of synaptic protein complexes using feature-guided alignment and 3D signal permutation using the same 80-pixel long by 40-pixel diameter cylindrical mask along with a 350 Å diameter spherical mask for RELION. Intermembrane densities emerge, situated between the membranes. Classes 1, 2, and 3 contain 419, 433, and 621 intermembrane densities, respectively. Class averages were filtered to the respective resolution calculated by post-processing—30.5 Å, 27.9 Å, and 33.5 Å for class 1, 2, and 3 respectively. Contour levels of normalized volumes are 6.33, 5.22, and 9.93 σ, respectively. **(D)** Cross correlation coefficient (CCC) value distributions for each of the classes. **(E)** Examples of class average maps from 3D classification/*v*-restrained refinement alone created from Class 2 subtomograms with CCC values >0 (left, 405 intermembrane complexes) and <0 (right, 216 intermembrane complexes) (See also Supplementary Figure 2). 3D classification/*v*-restrained refinement with RELION was performed for 25 iterations with three classes in **A** (increasing iterations increased noise) and 100 iterations with three classes in **B** and **C.** No symmetry was imposed, a 5-pixel offset range, a 1-pixel offset step, and a regularization parameter of 2 was used. Prior angles were provided for the “tilt” and “psi” angles, and these angles were limited to a 5° local search range, effectively imposing a restrained rotational search around the vector *v*. To restrict these angles, “_rlnAngleTilt” and “_rlnAnglePsi” values were added to the RELION STAR file as “_rlnAngleTiltPrior” and “_rlnAnglePsiPrior” respectively.

*Figure 7. CCC filtering, 3D signal permutation and mask optimization produces classes that converge after continued iterations.*

**(A)** Starting with the last step of the 3D classification/*v*-restrained refinement related to Figure 6C, only subtomograms with CCC>0 were kept, 3D signal permutation repeated with unique cylindrical masks generated for each class centered around the aligned coordinates, followed by *v*-restrained refinement, auto-refinement and post-processing. For class 1 (337 intermembrane complexes), we used an 80-pixel long by 50-pixel diameter mask. For class 2 (388 intermembrane complexes), we used an 80x40 pixel mask, and for class 3 (405 intermembrane complexes), we used a 90x40 pixel mask. Class averages after 3D classification followed by auto-refinement and post-processing of each class individually. 3D classification nearly reached convergence after 200 iterations, and there were only small changes between 200 and 300 iterations. No symmetry was imposed, a 1-pixel offset range with a 0.5-pixel offset step was used, and local searches from auto-sampling was set to 1.8°. A data-inspired mask with 3 pixels of extension and a 35-pixel soft edge was created in RELION for post- processing. Post-processed class averages were calculated with B-factor set to 200 Å^2^ and low-pass filtering at 15 Å. Class averages were filtered to the respective resolution calculated by post-processing—24.0 Å, 25.8 Å, and 30.5 Å for class 1, 2, and 3 respectively. Contour levels of normalized volumes are 5.76, 5.06, and 6.95 σ, respectively. **(B)** Averaged intermembrane densities after applying angular rotations obtained in (A) and without 3D signal permutation—only a 250 Å diameter spherical RELION mask was used (left). Overlay of the expanded class 1 interface with the class 1 average after postprocessing shown in panel (A) (right). Again, no symmetry was imposed, a 5-pixel offset range, a 1-pixel offset step and a regularization parameter of 2 was used in generating the class averages. Prior angles were provided for the “tilt” and “psi” angles, limited to 5° local search range.

**Supplementary Figures**

*Supplementary Figure 1. 3D classification of 3D-signal-permuted synaptic interfaces without intensity filtering.*

Class averages after 3D classification alone of 3D-signal-permuted synaptic interfaces without intensity filtering. The same 3D classification parameters were used as in the 3D classification/v-restrained refinement related to Figure 6C. Intermembrane densities are less visible without background filtering of high-intensity voxels (compared to Figure 6C). Classes 1, 2, and 3 contain 352, 406, and 742 intermembrane complexes, respectively. Contour levels of normalized volumes are 5.72, 2.76, and 4.11 σ, respectively.

*Supplementary Figure 2. Examples of individual subtomograms of intermembrane densities after 3D classification/v-restrained refinement alone* ***(A)*** *related to Figure 6E left (CCC >0) and* ***(B)*** *related to Figure 6E right (CCC <0).*

*Supplementary Figure 3. Angular difference between vector v of subtomograms as a function of defocus.*

(**A**) The difference between the vectors *v* of the same subvolumes aligned by either RELION subtomogram averaging or NovaCTF/CreSTA/RELION workflows at iteration 0 was calculated by measuring the angular distance between the vectors *v*. These data quantify the difference between panels Figure 5A and 5F, where an angular difference of 180 degrees, for example, indicates a flipping of interface between the two workflows. (**B**) ANOVA multiple comparison of the defocus groups shown in A. As the defocus approaches 0 there is a higher propensity for large rotational differences, and specifically rotations that result in an orthogonal orientation. Non-overlapping distributions indicate significance level of p<0.05. (**C** and **D**) The angular difference of vectors *v* between the 0^th^ iteration and 10^th^ iteration within the two workflows after feature-guided alignment. During feature-guided alignment, Euler angles Tilt and Psi were constrained with a search step of 3.5 degrees while the rotation angle around vector *v* was not constrained. As expected, none of the distributions are significantly different from one another. These data relate to the differences between figure 5K and 5L (C) and 5P and 5Q (D). (**E**) The angular difference of vectors *v* between the two averaging workflows at the 10^th^ iteration after feature-guided alignment, comparing Figures 5L and 5Q. (**F**) ANOVA multiple comparison test of the defocus again shows that as defocus approaches 0, larger angular differences of vector v between the two methods emerge.

*Supplementary Figure 4.* *FSC curves for the three post-processed class averages, class 1 (****A****), class 2 (****B****), and class 3 (****C****).* In black are FSC curves without 3D-signal permutation. In magenta, FSC curves with 3D-signal permutation without mask optimization. In green, FSC curves with 3D-signal permutation and mask optimization.

**Appendix A. Example cryoET data set: synaptic protein complexes**

Grids were imaged using an FEI Titan Krios equipped with an energy filter and Gatan K2 Summit camera using a pixel size of 2.62 Å. Tilt series from these grids were collected using a dose-symmetric tilt scheme (Hagen et al., 2017) from -60° to +60° with 3° steps and 1.0-6.0 µm defocus using serialEM (Mastronarde, 2005). The pixel size was 2.62 Å.

Synaptic protein complexes at synaptic vesicle fusion interfaces were formed by glutamatergic synaptic vesicles (ISV) isolated from whole mouse brain and synthetic plasma membrane mimic (SM) vesicles containing Stx1 and SNAP-25 (Leitz et al., manuscript in preparation*)*. Details of sample preparation, blotting and plunge freezing presented elsewhere (Leitz et al., in preparation). Briefly, synaptosomes were prepared from mouse whole brain tissue, hypoosmotically lysed, and glutamatergic ISVs were immunoisolated using paramagnetic beads, as described previously (Grønborg et al., 2010). ISVs were then gently, competitively, eluted by addition of a small peptide corresponding to the antibody epitope. For SM vesicles, the preparation protocol was similar to. Lai et al., 2017 with the exception that SM conversion was performed in bulk solution by addition of the following proteins with final concentrations of: 2 mM Munc-18, 20 nM NSF, 100 nM a-SNAP, 1 mM ATP, 1 mM MgCl and 0.5 mM TCEP. ISVs were first added to 10 nM gold fiducials, deposited on the grids and incubated for 15 minutes followed by aspiration of excess solution. The following proteins were added to the SM vesicle mixture with final concentrations of: 1 mM complexin, 1 mM SNAP-25 0.5 mM TCEP 5 mM MUN. and this mixture was immediately added to the grid and incubated for 5 minutes. The grids were then loaded on a Vitrobot (ThermoFisher) and prior to plunging excess sample was aspirated and a wash solution containing 1 mM complexin was added. G A total of 299 tomograms were collected.

For both the RELION subtomogram averaging and NovaCTF/CrESTA/RELION workflows, prior to tomographic reconstruction, raw tilt frames were motion corrected with MotionCor2 (Zheng et al., 2017). Motion-corrected frames were then reassembled into tilt stacks using drift_combine.py. Tomograms were aligned and reconstructed using ETOMO (IMOD; Mastronarde and Held, 2017) using standard parameters. Importantly, during fine alignment, poorly fitting fiducials were corrected until the residual error mean value and standard deviation was less than 2.0 and 1.5, respectively. If the residual error could not reach that level, the tomogram was discarded (note with IMOD version 4.11.6 the residual error mean and standard deviation units were changed and tomograms were only used if these values were below 0.2 and 0.15, respectively). Bin4 and bin1 tomograms were generated, gold fiducials were removed, and tomograms were 2D Gaussian-filtered before reconstructions with a z thickness of 1,000-1,500 (unbinned pixels) were generated for both bin 1 and 4.

*NovaCTF 3D CTF estimation and correction*

For the NovaCTF/CrESTA/RELION workflow, we used NovaCTF (Turoňová et al., 2017; Wan et al., 2020) to recalculate the tomographic reconstructions using the tilt alignments obtained from the initial alignments obtained with ETOMO. NovaCTF performs 3D CTF estimation and correction on the entire tomogram. Full 3D CTF correction, such as implemented in NovaCTF, is essential for this tilt series due to the greater than 1 µm defocus difference, or up to 100% error in defocus estimation, in the periphery of the tilt series.

For the synaptic protein cryoET data set, we found that when CTFFIND4 estimated high tilt images (tilts greater than +/- 20 degrees), the calculated values were sometimes far outside reasonable values, which in turn greatly diminished the quality of the tomogram produced by NovaCTF. Therefore, we allowed CTFFIND4 to find the defocus value of the first 15 images (-21 degrees to +21 degrees). We then computed the average and standard deviation of defocus values of these 15 images. For tilts higher than 20 degrees, we kept the CTFFIND4 defocus value if the value was within one standard deviation of the average, otherwise the value was replaced with the average of the 15 images.

For the CTF-corrected tomographic reconstructions generated by NovaCTF, the effect of the missing wedge still needs to be accounted for in subsequent refinement processes. We generated a synthetic single missing wedge volume for the tilt scheme using *tom_wedge.m* (Nickel et al., 2005), and this volume is designated as the “_rlnCtfImage” for each subtomogram in the STAR file inputted into subsequent RELION classification and refinement jobs (Figure 3C). In principle, this approach can be generalized to apply dose filtering (Wan et al., 2020).

*Global interface coordinate selection*

Two coordinates were manually selected for each interface in 3DMOD (Mastronarde and Held, 2017) using 4x binned tomographic reconstructions with inverted contrast such that feature-guided alignment could be performed. If multiple coordinates were selected for each object of interest, each object was saved as a single contour and the two coordinates saved as points within that contour. For example, for subtomogram averaging using the global interface between membranes (rather than individual protein interface densities, see section below), for each interface, a coordinate at the center of the ISV membrane at the fusion interface was selected as contour 1 point 1 and the center of the ISV for the same interface (referred to as “ISV center”) were picked and saved as contour 1, point 2 (Figure 1A). The next interface would then have the ISV membrane as contour 2, point 1 and the corresponding ISV center would be contour 2, point 2, and so on. ISVs were identified by the protruding density of the V-ATPases (Figure 3B, blue asterisk). The point 1 coordinates (centers of the ISV membrane) were used as the center coordinates for the subtomogram extraction box for the global interfaces (used in Figure 5). The vector *v* between the ISV membrane and ISV center represents an axis of interest for later alignment steps (see Figure 4C).

Once coordinates have been selected, they were saved as a .mod file. These were converted into x y z coordinates using mod2point (IMOD) or performed in batch using CreateCoords.sh. The resulting .coord file was parsed using Cordsort_batch.sh such that the coordinates of all contours are grouped together based on point number. That is, all the ISV membranes for all target locations were grouped into a single .coord file for each tomogram, and the ISV centers were similarly grouped into a separate .coord file.

*Initial subtomogram extractions*

For both the RELION subtomogram averaging and the NovaCTF/CrESTA/RELION workflows, the specified coordinates were used for subtomogram extraction using RELION. Subtomograms were normalized during extraction and were extracted with inverted contrast and a 256-pixel box size.

*Free alignment*

For both the RELION subtomogram averaging and the NovaCTF/CrESTA/RELION workflows, free alignment was performed as described in the Methods, and the subtomograms re-oriented to align vector *v* to the z-axis (Figure 1B).

*Feature-guided alignment*

For both the RELION subtomogram averaging and the NovaCTF/CrESTA/RELION workflows, feature-guided alignment was performed as described in the Methods to align vector *v* to the z-axis (Figure 1C).

*Intermembrane density coordinate selections and subtomogram extractions*

For subtomogram extractions around intermembrane densities with the NovaCTF/CrESTA/RELION workflow, individual protein interface densities in each subtomogram were visually inspected in Chimera and a Wiener filter with defocus values ranging from 1.0-6.0 and a signal-to-noise ratio falloff of 1.2 (Nickel et al., 2005) was applied for easier visualization of densities. A coordinate was selected for each membrane-spanning density, and the Euler angles from global interface alignment was used. In the general case, each object will need a second coordinate by which to create vector, *v*.

*3D signal permutation*

For the first round of 3D signal permutation, we used a 40-pixel diameter by 80-pixel long cylindrical mask. We used the following parameters for the signal permutation process. Extreme values (voxels with values greater than or less than 1.75 from the mean) were removed from the background and replaced with a random value selected from a normal distribution with a mean of 0. After extreme value removal, the location of background voxels was permuted with a blur rate of 1.1 to prevent the formation of sharp boundaries. During iterative steps of signal permutation, the above parameters were maintained, but unique cylindrical masks were generated for each class average.

*Repeat 3D signal permutation*

For the subsequent round of class-specific re-extraction we used three unique masks. For class 1, we used an 80 pixel long by 50-pixel diameter mask. For class 2, we used a 80x40 pixel mask, and for class 3, we used a 90x40 pixel mask. 3D signal permutation was centered around the coordinates of the aligned maps from the previous iteration of 3D signal permutation and subsequent 3D refinement in RELION.

**Appendix B. Parallel implementation of NovaCTF**

We implemented a parallelized implementation of NovaCTF similar to the STOPGAP implementation of NovaCTF (Figure 1) (Turoňová et al., 2017; Wan et al., 2020). Tomograms were constructed using a newly developed Nova_Updated.sh bash script that uses the NovaCTF pipeline with optional parallelization. Specifically, the script first performs defocus estimation using CTFFIND4 with a step size determined by the user, here we used 0.5 µm (Rohou and Grigorieff, 2015) and generates the defocus text file required for NovaCTF defocus algorithm. Next the NovaCTF algorithm CTF Correction is run with the .tlt file generated in Etomo. Here our pipeline then applies the alignments generated in ETOMO using IMOD commands and the fid.xf file. Next gold is similarly removed using the erase.fid file, the edges are tapered, if desired, and any views (i.e., specific bad tilt angle images) are removed using either the tilt.com file—which contains an exclusion list if bad images were excluded in the ETOMO initialization—or a user-generated exclusions.txt file containing a comma-separated list of images (specifically the image number, not the tilt angle of the image). Next the remaining NovaCTF steps are performed: y-z reorientation, filter projections algorithm and finally the 3dctf algorithm reconstructs the tomogram (Figure 1) (Turoňová et al., 2017; Wan et al., 2020).

**Appendix C. Implementation of the NovaCTF/CrESTA/RELION pipeline**

Below is the general strategy for reconstructing and CTF correcting tomograms, extracting and aligning subtomograms, 3D signal permutation from subtomograms, classifying subtomograms, manually and systematically forming subsets from initial classes, and refining class averages. Included are the necessary software. A summary of the data processing pipeline with details of the scripts is shown in Figure 4B. A schematic of subtomogram extraction and feature-guided alignment is shown in Figure 1. An example of the CrESTA GUI is shown in Figure 2. The CrESTA package is available on GitHub (<https://github.com/johnjacobpeters/tom_cryoET>).

*Tomogram reconstruction, subtomogram picking, extraction, and 3D CTF correction*

1. Motion correct raw frames with MotionCor2 and reassemble frames into tilt stacks using *drift_combine.py*, or in batch using one of the custom scripts BatchDriftCombine_K2/3, depending on the microscope camera.
2. Reconstruct tomograms using ETOMO (IMOD). The minimum files that must be generated are the .ali, .tlt, erase.fid, fid.xf, tilt.com and an optional exclusions.txt containing frames to be excluded if not already specified during tomogram generation in ETOMO. Additionally, the .mdoc file is required.
3. Pick coordinates by inspection in 3DMOD (IMOD). If multiple coordinates per object of interest are selected, the coordinates will need to be parsed into each group. As an example, see the *PrepareCoords.sh* bash script which generate folders for each tomogram with appropriate file types and renamed extensions.
4. Reconstruct tomograms using Nova_Updated.sh bash script. The script creates a defocus array using defocus values obtained from CTFFIND4 (Rohou and Grigorieff, 2015), creates and applies a defocus matrix using NovaCTF (Turanova et al.,). The tilt images are aligned, gold erased, and tomograms are reconstructed using information generated during the initial reconstruction in ETOMO (step 3).
5. Generate synthetic missing wedge volumes using *tom_wedge.m* (Nickel et al., 2005) to account for missing wedge artifact. This volume is designated as the “_rlnCtfImage” for each subtomogram in the STAR file.
6. Extract subtomograms with RELION with inverted contrast and normalization. For objects of interest with a continuous feature (e.g., lipid bilayers) the inclusion of this feature in early steps can lead to improved alignment of subtomograms. Note that switching between the NovaCTF-corrected and uncorrected tomographic reconstructions from extraction can be done by simply choosing which reconstruction is used for the extraction.

*Alignment of subtomograms*

1. Single-class alignment: To use RELION to align subtomograms, perform 3D refinement with one class. To orient the resulting class average to a desired orientation, use the “Display mrc in tom xyz” tool on the visualization tab of CrESTA (Figure 2B).

OR

1. Feature-guided alignment: Alternatively, if your object of interest is difficult to align or if you have additional context for your object of interest, select two coordinates per object and use the python script *transform_project.py* to perform feature-guided alignment of interfaces along an axis of interest.

3*D signal permutation from subtomograms*

1. Perform 3D signal permutation on subtomograms using CrESTA (Figure 2B). CrESTA generates a new STAR file identified by the *reextract.star* extension that can be used for classification within RELION. During 3D signal permutation, background filtering is essential to reduce the contribution of high intensity voxels in the background. Either standard deviation shifting or random noise generation can be used to filtering the background. Some blurring between the mask and the background can help reduce potential artifacts introduced by 3D signal permutation.
2. Further refinement of class averages using RELION should be performed after 3D signal permutation.
3. After refinement, perform a new 3D signal permutation from the original subtomograms using the refined translations and rotations to correct for any subtle mistakes in the manual picking process. Also, unique geometric masks for each class can be generated and used at this stage. Perform further refinements in RELION with these corrected subtomograms. The results of refinement can be visualized using the “Stackbrowser” tool in CrESTA for manual subset selection. Additionally, the cross-correlation module (which uses *tom_corr_wedge.m)* in CrESTA can be used to systematically filter out poor-fit subtomograms. Further refinement of classes using RELION should be performed after pruning.
4. Iterate step 8 as many times as desired to improve class averages.

*Necessary equipment and software for NovaCTF/CrESTA/RELION workflow*

Equipment

- A CPU and/or GPU cluster. GPUs greatly reduces processing time in RELION.

Software

- MATLAB
- RELION 3 (Scheres, 2012; Bharat et al., 2015; Bharat and Scheres, 2016)
- Python3
- NovaCTF (Turoňová et al., 2017; Wan et al., 2020)
- IMOD (Mastronarde and Held, 2017)
- Chimera (Pettersen et al., 2004)
- CrESTA
- TOM Toolbox (Nickel et al., 2005)

**References**

**﻿**Bammes, B. E., Rochat, R. H., Jakana, J., Chen, D.-H., & Chiu, W. (2012). Direct electron detection yields cryo-EM reconstructions at resolutions beyond 3/4 Nyquist frequency. *Journal of Structural Biology*, 177(3), 589–601. https://doi.org/https://doi.org/10.1016/j.jsb.2012.01.008

﻿Bepler, T., Morin, A., Rapp, M., Brasch, J., Shapiro, L., Noble, A. J., & Berger, B. (2019). Positive-unlabeled convolutional neural networks for particle picking in cryo-electron micrographs. *Nature Methods*, 16(11), 1153–1160. https://doi.org/10.1038/s41592-019-0575-8

﻿Bharat, T. A. M., Russo, C. J., Löwe, J., Passmore, L. A., & Scheres, S. H. W. (2015). Advances in Single-Particle Electron Cryomicroscopy Structure Determination applied to Sub-tomogram Averaging. *Structure*, 23(9), 1743–1753. https://doi.org/10.1016/j.str.2015.06.026

﻿Bharat, T. A. M., & Scheres, S. H. W. (2016). Resolving macromolecular structures from electron cryo-tomography data using subtomogram averaging in RELION. *Nature Protocols*, 11(11), 2054–2065. https://doi.org/10.1038/nprot.2016.124

﻿Bouvette, J., Liu, H.-F., Du, X., Zhou, Y., Sikkema, A. P., da Fonseca Rezende e Mello, J., Klemm, B. P., Huang, R., Schaaper, R. M., Borgnia, M. J., & Bartesaghi, A. (2021). Beam image-shift accelerated data acquisition for near-atomic resolution single-particle cryo-electron tomography. *Nature Communications*, *12*(1), 1957. https://doi.org/10.1038/s41467-021-22251-8

﻿Briggs, J. A. G., Riches, J. D., Glass, B., Bartonova, V., Zanetti, G., & Kräusslich, H. G. (2009). Structure and assembly of immature HIV. *Proceedings of the National Academy of Sciences of the United States of America*, 106(27), 11090–11095. https://doi.org/10.1073/PNAS.0903535106

﻿Chen, M., Bell, J. M., Shi, X., Sun, S. Y., Wang, Z., & Ludtke, S. J. (2019). A complete data processing workflow for cryo-ET and subtomogram averaging. *Nature Methods*, 16(11), 1161–1168. https://doi.org/10.1038/s41592-019-0591-8

﻿Chreifi, G., Chen, S., Metskas, L. A., Kaplan, M., & Jensen, G. J. (2019). Rapid tilt-series acquisition for electron cryotomography. *Journal of Structural Biology*, 205(2), 163–169. https://doi.org/https://doi.org/10.1016/j.jsb.2018.12.008

﻿Chreifi, G., Chen, S., & Jensen, G. J. (2021). Rapid tilt-series method for cryo-electron tomography: Characterizing stage behavior during FISE acquisition. *Journal of Structural Biology*, 213(2), 107716. https://doi.org/https://doi.org/10.1016/j.jsb.2021.107716

Eisenstein, M. (2016). The field that came in from the cold. *Nature Methods*, 13(1), 19–22. https://doi.org/10.1038/nmeth.3698

﻿Eisenstein, F., Danev, R., & Pilhofer, M. (2019). Improved applicability and robustness of fast cryo-electron tomography data acquisition. *Journal of Structural Biology*, 208(2), 107–114. https://doi.org/https://doi.org/10.1016/j.jsb.2019.08.006

﻿Galaz-Montoya, J. G., Flanagan, J., Schmid, M. F., & Ludtke, S. J. (2015). Single particle tomography in EMAN2. *Journal of Structural Biology*, 190(3), 279–290. https://doi.org/https://doi.org/10.1016/j.jsb.2015.04.016

﻿Galaz-Montoya, J. G., Hecksel, C. W., Baldwin, P. R., Wang, E., Weaver, S. C., Schmid, M. F., Ludtke, S. J., & Chiu, W. (2016). Alignment algorithms and per-particle CTF correction for single particle cryo-electron tomography. *Journal of Structural Biology*, 194(3), 383–394. https://doi.org/https://doi.org/10.1016/j.jsb.2016.03.018

﻿Glaeser, R. M., & Han, B.-G. (2017). Opinion: hazards faced by macromolecules when confined to thin aqueous films. *Biophysics Reports*, 3(1), 1–7. https://doi.org/10.1007/s41048-016-0026-3

﻿Grant, T., Rohou, A., & Grigorieff, N. (2018). cisTEM, user-friendly software for single-particle image processing. *ELife*, 7, e35383. https://doi.org/10.7554/eLife.35383

Grønborg, M., Pavlos, N. J., Brunk, I., Chua, J. J. E., Münster-Wandowski, A., Riedel, D., Ahnert-Hilger, G., Urlaub, H., & Jahn, R. (2010). Quantitative Comparison of Glutamatergic and GABAergic Synaptic Vesicles Unveils Selectivity for Few Proteins Including MAL2, a Novel Synaptic Vesicle Protein. *The Journal of Neuroscience*, 30(1), 2 LP – 12. https://doi.org/10.1523/JNEUROSCI.4074-09.2010

﻿Hagen, W. J. H., Wan, W., & Briggs, J. A. G. (2017). Implementation of a cryo-electron tomography tilt-scheme optimized for high resolution subtomogram averaging. *Journal of Structural Biology*, 197(2), 191–198. https://doi.org/https://doi.org/10.1016/j.jsb.2016.06.007

﻿Henderson, R. (2015). Overview and future of single particle electron cryomicroscopy. *Archives of Biochemistry and Biophysics*, 581, 19–24. https://doi.org/https://doi.org/10.1016/j.abb.2015.02.036

﻿Heumann, J. M., Hoenger, A., & Mastronarde, D. N. (2011). Clustering and variance maps for cryo-electron tomography using wedge-masked differences. *Journal of Structural Biology*, 175(3), 288–299. https://doi.org/https://doi.org/10.1016/j.jsb.2011.05.011

﻿Himes, B. A., & Zhang, P. (2018). emClarity: software for high-resolution cryo-electron tomography and subtomogram averaging. *Nature Methods*, 15(11), 955–961. https://doi.org/10.1038/s41592-018-0167-z

﻿Hrabe, T., Chen, Y., Pfeffer, S., Kuhn Cuellar, L., Mangold, A.-V., & Förster, F. (2012). PyTom: A python-based toolbox for localization of macromolecules in cryo-electron tomograms and subtomogram analysis. *Journal of Structural Biology*, 178(2), 177–188. https://doi.org/https://doi.org/10.1016/j.jsb.2011.12.003

﻿

Lai, Y., Choi, U. B., Leitz, J., Rhee, H. J., Lee, C., Altas, B., Zhao, M., Pfuetzner, R. A., Wang, A. L., Brose, N., Rhee, J. S., & Brunger, A. T. (2017). Molecular Mechanisms of Synaptic Vesicle Priming by Munc13 and Munc18. *Neuron*, 95(3), 591-607.e10. https://doi.org/10.1016/J.NEURON.2017.07.004

﻿Mastronarde, D. N. (2005). Automated electron microscope tomography using robust prediction of specimen movements. *Journal of Structural Biology*, 152(1), 36–51. https://doi.org/https://doi.org/10.1016/j.jsb.2005.07.007

﻿Mastronarde, D. N., & Held, S. R. (2017). Automated tilt series alignment and tomographic reconstruction in IMOD. *Journal of Structural Biology*, 197(2), 102–113. https://doi.org/https://doi.org/10.1016/j.jsb.2016.07.011

﻿McMullan, G., Faruqi, A. R., Clare, D., & Henderson, R. (2014). Comparison of optimal performance at 300keV of three direct electron detectors for use in low dose electron microscopy. *Ultramicroscopy*, 147, 156–163. https://doi.org/https://doi.org/10.1016/j.ultramic.2014.08.002

﻿

Milazzo, A.-C., Leblanc, P., Duttweiler, F., Jin, L., Bouwer, J. C., Peltier, S., Ellisman, M., Bieser, F., Matis, H. S., Wieman, H., Denes, P., Kleinfelder, S., & Xuong, N.-H. (2005). Active pixel sensor array as a detector for electron microscopy. *Ultramicroscopy*, 104(2), 152–159. https://doi.org/https://doi.org/10.1016/j.ultramic.2005.03.006

﻿ ﻿Morais, M. C., Kanamaru, S., Badasso, M. O., Koti, J. S., Owen, B. A. L., McMurray, C. T., Anderson, D. L., & Rossmann, M. G. (2003). Bacteriophage φ29 scaffolding protein gp7 before and after prohead assembly. *Nature Structural & Molecular Biology*, 10(7), 572–576. https://doi.org/10.1038/nsb939

Nakane, T., Kimanius, D., Lindahl, E., & Scheres, S. H. W. (2018). Characterisation of molecular motions in cryo-EM single-particle data by multi-body refinement in RELION. *ELife*, 7, e36861. https://doi.org/10.7554/eLife.36861

Nakane, T., Kotecha, A., Sente, A., McMullan, G., Masiulis, S., Brown, P. M. G. E., Grigoras, I. T., Malinauskaite, L., Malinauskas, T., Miehling, J., Uchański, T., Yu, L., Karia, D., Pechnikova, E. V, de Jong, E., Keizer, J., Bischoff, M., McCormack, J., Tiemeijer, P., … Scheres, S. H. W. (2020). Single-particle cryo-EM at atomic resolution. *Nature*, 587(7832), 152–156. https://doi.org/10.1038/s41586-020-2829-0﻿

﻿Navarro, P. P., Stahlberg, H., & Castaño-Díez, D. (2018). Protocols for Subtomogram Averaging of Membrane Proteins in the Dynamo Software Package. *Frontiers in Molecular Biosciences* (Vol. 5, p. 82). https://www.frontiersin.org/article/10.3389/fmolb.2018.00082

Nickell, S., Förster, F., Linaroudis, A., Net, W. Del, Beck, F., Hegerl, R., Baumeister, W., & Plitzko, J. M. (2005). TOM software toolbox: acquisition and analysis for electron tomography. *Journal of Structural Biology*, 149(3), 227–234. https://doi.org/https://doi.org/10.1016/j.jsb.2004.10.006

Nogales, E. (2016). The development of cryo-EM into a mainstream structural biology technique. *Nature Methods*, 13(1), 24–27. https://doi.org/10.1038/nmeth.3694

﻿Pettersen, E. F., Goddard, T. D., Huang, C. C., Couch, G. S., Greenblatt, D. M., Meng, E. C., & Ferrin, T. E. (2004). UCSF Chimera—A visualization system for exploratory research and analysis. Journal of Computational Chemistry, 25(13), 1605–1612. https://doi.org/https://doi.org/10.1002/jcc.20084

﻿Punjani, A., Rubinstein, J. L., Fleet, D. J., & Brubaker, M. A. (2017). cryoSPARC: algorithms for rapid unsupervised cryo-EM structure determination. *Nature Methods*, 14(3), 290–296. https://doi.org/10.1038/nmeth.4169

﻿Rohou, A., & Grigorieff, N. (2015). CTFFIND4: Fast and accurate defocus estimation from electron micrographs. *Journal of Structural Biology*, 192(2), 216–221. https://doi.org/https://doi.org/10.1016/j.jsb.2015.08.008

﻿Scheres, S. H. W. (2012). RELION: Implementation of a Bayesian approach to cryo-EM structure determination. *Journal of Structural Biology*, 180(3), 519–530. https://doi.org/https://doi.org/10.1016/j.jsb.2012.09.006

﻿

Shuster, M. D., & Markley, F. L. (2006). General Formula for Extracting the Euler Angles. *Journal of Guidance, Control, and Dynamics*, 29(1), 215–221. https://doi.org/10.2514/1.16622

Stalling, D., Westerhoff, M., Hege. H.-C., (2005) Amira: a highly interactive system for visual data analysis. *C.D. Hansen, C.R. Johnson (Eds.), The Visualization Handbook, Elsevier Butterworth-Heinemann (2005),* pp. 749-768

﻿

﻿Tegunov, D., & Cramer, P. (2019). Real-time cryo-electron microscopy data preprocessing with Warp. *Nature Methods*, 16(11), 1146–1152. https://doi.org/10.1038/s41592-019-0580-y

﻿Tegunov, D., Xue, L., Dienemann, C., Cramer, P., & Mahamid, J. (2021). Multi-particle cryo-EM refinement with M visualizes ribosome-antibiotic complex at 3.5 Å in cells. *Nature Methods*, 18(2), 186–193. https://doi.org/10.1038/s41592-020-01054-7

**﻿**Turoňová, B., Schur, F. K. M., Wan, W., & Briggs, J. A. G. (2017). Efficient 3D-CTF correction for cryo-electron tomography using NovaCTF improves subtomogram averaging resolution to 3.4Å. *Journal of Structural Biology*, 199(3), 187–195. https://doi.org/https://doi.org/10.1016/j.jsb.2017.07.007

﻿Turoňová, B., Sikora, M., Schürmann, C., Hagen, W. J. H., Welsch, S., Blanc, F. E. C., von Bülow, S., Gecht, M., Bagola, K., Hörner, C., van Zandbergen, G., Landry, J., de Azevedo, N. T. D., Mosalaganti, S., Schwarz, A., Covino, R., Mühlebach, M. D., Hummer, G., Krijnse Locker, J., & Beck, M. (2020). In situ structural analysis of SARS-CoV-2 spike reveals flexibility mediated by three hinges. *Science*, 370(6513), 203 LP – 208. https://doi.org/10.1126/science.abd5223

Wan, W., Khavnekar, S., Wagner, J., Erdmann, P., & Baumeister, W. (2020). STOPGAP: A Software Package for Subtomogram Averaging and Refinement. *Microscopy and Microanalysis*, 26(S2), 2516-2516. doi:10.1017/S143192762002187X

﻿Zhang, P. (2019). Advances in cryo-electron tomography and subtomogram averaging and classification. *Current Opinion in Structural Biology*, 58, 249–258. https://doi.org/https://doi.org/10.1016/j.sbi.2019.05.021

﻿Zheng, S. Q., Palovcak, E., Armache, J.-P., Verba, K. A., Cheng, Y., & Agard, D. A. (2017). MotionCor2: anisotropic correction of beam-induced motion for improved cryo-electron microscopy. *Nature Methods*, 14(4), 331–332. https://doi.org/10.1038/nmeth.4193
