# Supplementary figures and images for: A feature-guided, focused 3D signal permutation method for subtomogram averaging
Source: J Struct Biol. Author manuscript; Available in PMC 2022 Jun 1. (PMC9149098; doi:10.1016/j.jsb.2022.107851)

Class 1

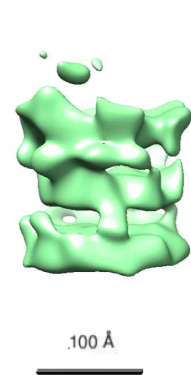

Class 2

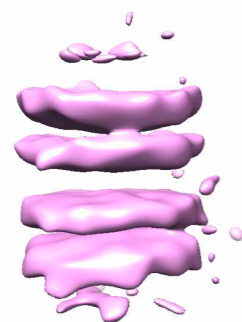

Class 3

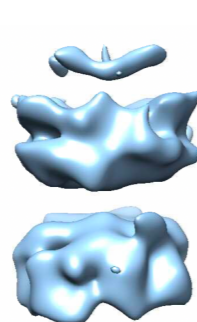

Supplement: 2 [file NIHMS1795382-supplement-2.pdf]

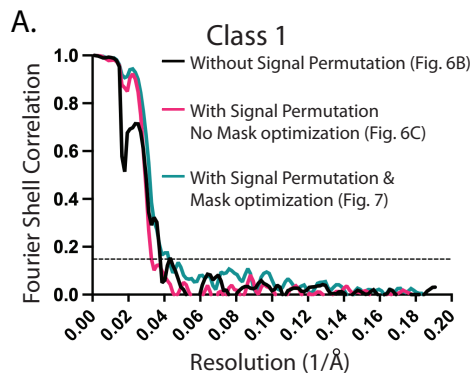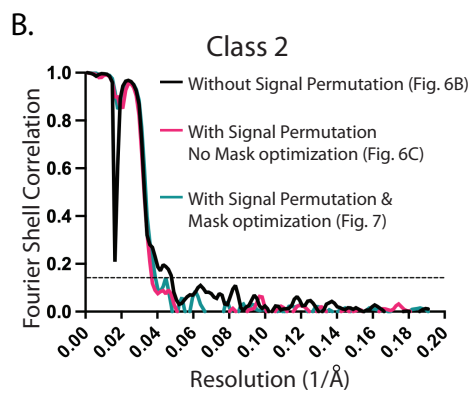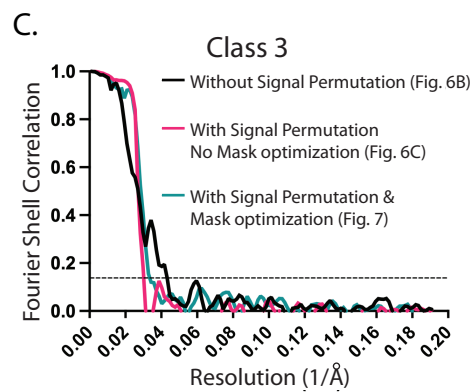

Supplement: 5 [file NIHMS1795382-supplement-5.pdf]
