# Supplementary material for: A feature-guided, focused 3D signal permutation method for subtomogram averaging
Source: J Struct Biol. Author manuscript; Available in PMC 2022 Jun 1. (PMC9149098; doi:10.1016/j.jsb.2022.107851)
Supplement: 3 [file NIHMS1795382-supplement-3.pdf]

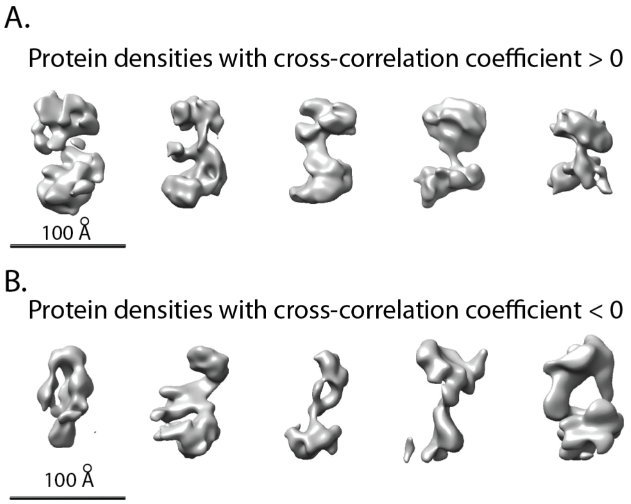
A.
Protein densities with cross-correlation coefficient > 0
100 Å
B.
Protein densities with cross-correlation coefficient < 0
100 Å
