# Supplementary material for: A feature-guided, focused 3D signal permutation method for subtomogram averaging
Source: J Struct Biol. Author manuscript; Available in PMC 2022 Jun 1. (PMC9149098; doi:10.1016/j.jsb.2022.107851)
Supplement: 4 [file NIHMS1795382-supplement-4.pdf]

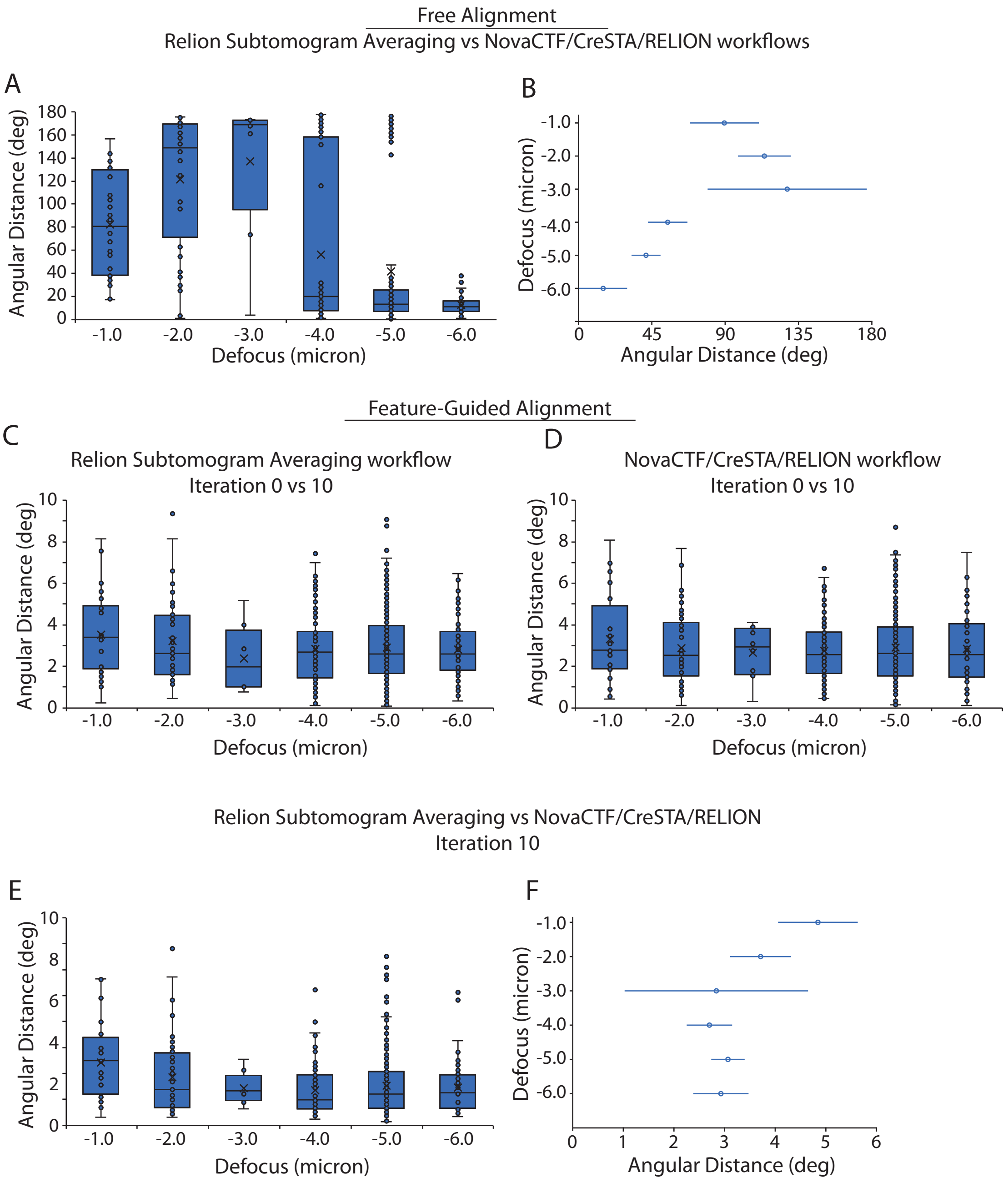
Free Alignment
Relion Subtomogram Averaging vs NovaCTF/CreSTA/RELION workflows
A
Angular Distance (deg)
180
160
140
120
100
80
60
40
20
0
-1.0
-2.0
-3.0
-4.0
-5.0
-6.0
Defocus (micron)
B
Defocus (micron)
-1.0
-2.0
-3.0
-4.0
-5.0
-6.0
0
45
90
135
180
Angular Distance (deg)
Feature-Guided Alignment
C
Relion Subtomogram Averaging workflow
Iteration 0 vs 10
Angular Distance (deg)
Defocus (micron)
D
NovaCTF/CreSTA/RELION workflow
Iteration 0 vs 10
Angular Distance (deg)
Defocus (micron)
Relion Subtomogram Averaging vs NovaCTF/CreSTA/RELION
Iteration 10
E
Angular Distance (deg)
Defocus (micron)
F
Defocus (micron)
0
1
2
3
4
5
6
Angular Distance (deg)
